# Dasatinib-Induced Nephrotic Syndrome: A Case Report

**DOI:** 10.7759/cureus.20330

**Published:** 2021-12-10

**Authors:** Ahmed ElShaer, Mazen Almasry, Maher Alawar, Hassan Masoud, Abdul Rahman El Kinge

**Affiliations:** 1 Internal Medicine, Alfaisal University College of Medicine, Riyadh, SAU; 2 Nephrology, Specialized Medical Center, Riyadh, SAU; 3 Pathology, Specialized Medical Center, Riyadh, SAU; 4 Hematology and Medical Oncology, NMC Royal Hospital, Sharjah, ARE

**Keywords:** case report, adverse event, proteinuria, tyrosine kinase inhibitor, renal disease

## Abstract

Second-generation tyrosine kinase inhibitors (TKI), such as nilotinib and dasatinib, are used in the first-line treatment of chronic myeloid leukemia (CML), usually after the failure or resistance to imatinib. Despite a good safety profile, medications in this category have an increased incidence of specific adverse events such as pulmonary hypertension, pleural effusion, and cardiovascular/peripheral arterial events. However, renal complications are rarely reported and observed. We herein report a case of a 46-year-old patient with CML who developed nephrotic syndrome upon switching from imatinib to dasatinib therapy, with the resolution of symptoms upon treatment discontinuation and switching to nilotinib. Limited cases were reported in the literature. It is thought that the inhibition of the vascular endothelial growth factor (VEGF) pathway is the main mechanism leading to proteinuria. Dasatinib-induced nephrotic syndrome should be looked for as it can be resolved by either reducing the dose or stopping it altogether and switching to another TKI.

## Introduction

Chronic myelogenous leukemia (CML) is a hematological malignancy that accounts for almost 20% of leukemia cases in adults [1]. CML is characterized by a reciprocal t(9,22) translocation resulting in the fusion of BCR-ABL. This genetic abnormality, which continuously activates tyrosine kinase (TK) resulting in leukemia-cell proliferation, is the major mechanism behind the development of the disease [2]. The discovery of t(9,22) translocation pathogenesis led to a revolution in the treatment of CML as it paved the way to the development and approval of tyrosine kinase inhibitors (TKIs). Imatinib is a first-generation tyrosine kinase inhibitor (TKI) used in the treatment of chronic myeloid leukemia (CML) [3]. Although imatinib has a good safety profile, 30% of patients develop primary or secondary resistance, which led to the development of second-generation TKIs, namely dasatinib and nilotinib [4-7]. Several side-effects of special interest were reported with second-generation TKIs including pulmonary hypertension, pleural effusion, and cardiovascular/peripheral arterial events [8, 9]. However, renal complications are less commonly reported and observed. We herein report a case of a 46-year-old patient with chronic myelogenous leukemia who developed nephrotic syndrome upon switching from imatinib to dasatinib therapy, showing a resolution of symptoms upon treatment discontinuation, and switching to nilotinib. Similar cases in the literature are reviewed with inhibition of the vascular endothelial growth factor (VEGF) pathway thought to be the main mechanism leading to proteinuria. Dasatinib-induced nephrotic syndrome can be resolved by either reducing the dose or stopping dasatinib and switching to another TKI.

## Case presentation

**Figure 1:**
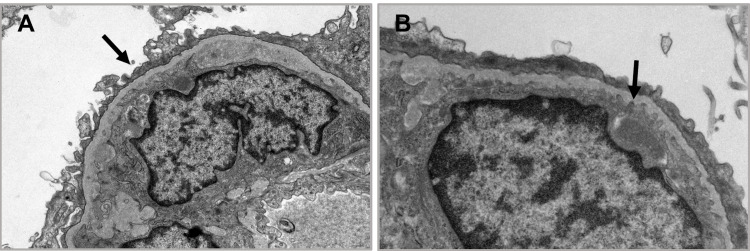
(A) Podocytes display moderate foot process effacement involving approximately 50% of the total peripheral capillary surface area. (B) Arrow pointing at the accumulation of lipid droplets within the subendothelial zone.

A 46-year-old female patient was diagnosed with Philadelphia chromosome-positive chronic-phase chronic myeloid leukemia (Ph+ CML) in 2004, and she was maintained on imatinib mesylate 400 mg daily. In 2011, she had a loss of molecular and cytogenetic remission on imatinib. Accordingly, her treatment was switched to dasatinib 100 mg daily which she could not tolerate due to severe diffuse bone pain. The dose was reduced until the bone pain disappeared and she was maintained on 50 mg daily. After a year of therapy, she achieved major molecular response with undetectable BCR-ABL levels. However, five years later, while still in major molecular response, she presented with peculiar complaints. She had progressive periorbital puffiness, abdominal fullness, and shortness of breath on exertion. Physical examination revealed bilateral lower limb pitting edema +2, bilateral decreased air entry mainly on the left side, and hepatomegaly. Urinalysis showed 3+ proteinuria with epithelial cells. The spot urine protein to creatinine ratio was 5.33 mg/g which indicated nephrotic-range proteinuria without hematuria and with a normal creatinine of 0.79 mg/dL. Her 24-hour-urinary-protein test was collected and found to be 5.9 g/dL. In addition, the patient underwent serological testing of antinuclear antibody, anti-double-stranded DNA antibodies, and viral hepatitis which were all negative. Thereafter, the patient was evaluated by a nephrologist. A kidney biopsy was performed to reach a diagnosis. The findings of the biopsy on light microscopy showed normal cellularity with patent peripheral capillaries. However, there was segmental duplication in some glomeruli and few glomeruli had hyaline-type subendothelial and intramembranous deposits. The tubules showed mild acute injury with luminal ectasia and epithelial simplification. Tubular cells contained intracytoplasmic protein resorption droplets. The interstitium had no signs of pathology. The arteries and arterioles were normal with no acute thrombi. Electron microscopy showed endothelial cells segmentally swollen with loss of fenestrations with moderate effacement of the foot processes on the podocyte (Figure 1A). Accumulation of lipid droplets within the subendothelial zone was also noted (Figure 1B). The immunofluorescence study revealed no positive results for anti-IgA, IgG, IgM, C1q, C3, albumin, fibrinogen, kappa, and lambda. One month after discontinuing dasatinib, urinalysis showed 1+ proteinuria, spot urine protein to creatinine ratio decreased to 0.61 mg/g and total urine protein decreased to 822 mg/L. At the two-month follow-up, urinalysis showed 1+ proteinuria, urine protein to creatinine ratio further decreased to 0.31 mg/g and the total urine protein was 436 mg/L. The patient was switched to nilotinib 200 mg twice daily where she remained in major molecular response to date.

## Discussion

Nephrotic syndrome is a renal disease that occurs due to damage to the podocytes leading to a disruption in the glomerular filtration membrane. This results in hypercholesterolemia and proteinuria causing peripheral edema. Nephrotic syndrome is primarily caused by diseases of the glomerulus or as a secondary effect to a systematic disease [10].

Dasatinib, a second-generation TKI, is used to treat Ph+ CML and acute lymphoblastic leukemia (ALL), in addition to a spectrum of solid tumors that display c-Kit [11]. Although this drug is metabolized through the liver, nephrotic syndrome has been reported as a potential side effect in rare cases [12]. In phase 1 clinical trial that focused on dose-escalation and pharmacokinetics of dasatinib as a treatment for advanced solid tumors, 18% of participants developed proteinuria [13]. This adverse event is unrecognized as a potentially serious one and is possibly ignored. To the best of our knowledge, eight case reports of dasatinib-induced nephrotic syndrome were reported in the literature in patients with CML [10, 11, 14-19], making this present case, to our knowledge, the ninth case. In all CML cases, the major molecular response was maintained after either switching to another TKI or reducing the dose of dasatinib. Some proposed that reducing the dose by half was enough to resolve the proteinuria [19]. This indicates that the severity of proteinuria may be dose-dependent. The main clinical data of our case together with those in the literature are illustrated in Table 1.

**Table 1:** Reported cases of Dasatinib-induced nephrotic syndrome

Case	Ref	Patient	Duration of dasatinib	Urinary proteins excretion	Creatinine (mg/dl)	BCR-ABL ratio at dasatinib discontinuation	Duration to resolution	BCR-ABL ratio at last follow-up	Renal biopsy	Treatment	Prognosis
1	De Luca et al. [10]	45, F	6 months	4.0 g/day	0.9	2.67	2 weeks	0.036	NA	Switch to imatinib	Remission
2	Ruebner et al. [11]	3, F	17 months	UP/Ucr = 17g/gCr	0.3	NA	2 months	Negative	Focal foot process effacement	Discontinue	Remission
3	Wallace et al. [14]	63, F	3 months	3.9 g/day	0.79	NA	2 weeks	negative	Focal foot process effacement	Switch to imatinib	Remission
4	Ochiai et al. [15]	40, M	3 months	5.7 g/day	0.87	NA	2 weeks	NA	Endothelial cell injury and foot process effacement	Switch to nilotinib	Remission
5	Koinuma et al. [16]	52, F	5 years	UP/Ucr=2.18 g/gCr	0.83	NA	3 weeks	NA	Focal podocyte foot process effacement, and segmental endothelial cell swelling with a slight expansion of the subendothelial space	Switch to bosutinib	Remission
6	Piscitani et al. [17]	43, F	17 months	7.63 g/day	0.9	NA	5 months	NA	diffuse foot process effacement over the entire capillary surface	None	Unknown
7	Stanchina et al. [18]	53, M	< 10 days	10 g/day	NA	NA	3 days	NA	NA	Switch to imatinib	unknown
8	Mandac Rogulj et al. [19]	33, F	2 years	NA	NA	NA	NA	NA	NA	Dose reduction	Remission
9	Our case	46, F	5 years	UP/Ucr=5.33 g/gCr	0.60	0.02%	1 week	0.004%	Endothelial cells segmentally swollen with loss of fenestrations with moderate effacement of the foot processes on the podocyte	Switch to nilotinib	Remission

The safety profile of different TKIs varies according to the specific pathway of inhibition. For instance, blocking TKs such as c-abl, platelet-derived growth factor receptor, and epidermal growth factor receptor have shown beneficial outcomes on diseased kidneys in murine models [14]. On the other hand, the blockage of TKs in signaling pathways involving vascular endothelial growth factor (VEGF) can be damaging to the kidney, and hence, affecting its function [20]. The exact mechanism behind dasatinib-induced proteinuria remains obscure. The most acceptable theory suggests that blocking VEGF is the main mechanism behind dasatinib-induced proteinuria. VEGFs are known to play a central role in podocyte cytoskeletal organization [21]. The importance of VEGF in the development and function of glomerular endothelium was established by knocking out the VEGF gene from podocytes in a mouse model. As a result, this led to the development of proteinuria and hypertension in all mice [20]. Dasatinib inhibits Src family kinases which regulate the VEGF pathway in podocytes. Hence, it might induce proteinuria as seen in our case [21-24]. In contrast, nilotinib is thought to be renal protective and can prolong survival in patients with chronic kidney disease [25].

## Conclusions

In summary, nephrotic syndrome is a rare side effect that can be seen in patients treated with dasatinib. Inhibiting the VEGF pathway by dasatinib is thought to be the main mechanism leading to proteinuria. Dasatinib-induced nephrotic syndrome can be resolved by either reducing the dose or stopping dasatinib and switching to another TKI.
